# Identification of Knee Osteoarthritis Based on Bayesian Network: Pilot Study

**DOI:** 10.2196/13562

**Published:** 2019-07-18

**Authors:** Bo Sheng, Liang Huang, Xiangbin Wang, Jie Zhuang, Lihua Tang, Chao Deng, Yanxin Zhang

**Affiliations:** 1 College of Rehabilitation Medicine Fujian University of Traditional Chinese Medicine Fujian China; 2 Department of Mechanical Engineering The University of Auckland Auckland New Zealand; 3 Department of Exercise Sciences The University of Auckland Auckland New Zealand; 4 School of Kinesiology Shanghai University of Sport Shanghai China; 5 School of Mechanical Science and Engineering Huazhong University of Science and Technology Wuhan China

**Keywords:** osteoarthritis, knee, classification, health services for the aged, physical fitness, Bayesian network

## Abstract

**Background:**

Early identification of knee osteoarthritis (OA) can improve treatment outcomes and reduce medical costs. However, there are major limitations among existing classification or prediction models, including abstract data processing and complicated dataset attributes, which hinder their applications in clinical practice.

**Objective:**

The aim of this study was to propose a Bayesian network (BN)–based classification model to classify people with knee OA. The proposed model can be treated as a prescreening tool, which can provide decision support for health professionals.

**Methods:**

The proposed model’s structure was based on a 3-level BN structure and then retrained by the Bayesian Search (BS) learning algorithm. The model’s parameters were determined by the expectation-maximization algorithm. The used dataset included backgrounds, the target disease, and predictors. The performance of the model was evaluated based on classification accuracy, area under the curve (AUC), specificity, sensitivity, positive predictive value (PPV), and negative predictive value (NPV); it was also compared with other well-known classification models. A test was also performed to explore whether physical fitness tests could improve the performance of the proposed model.

**Results:**

A total of 249 elderly people between the ages of 60 and 80 years, living in the Kongjiang community (Shanghai), were recruited from April to September 2007. A total of 157 instances were adopted as the dataset after data preprocessing. The experimental results showed that the results of the proposed model were higher than, or equal to, the mean scores of other classification models: .754 for accuracy, .78 for AUC, .78 for specificity, and .73 for sensitivity. The proposed model provided .45 for PPV and .92 for NPV at the prevalence of 20%. The proposed model also showed a significant improvement when compared with the traditional BN model: 6.3% increase in accuracy (from .709 to .754), 4.0% increase in AUC (from .75 to .78), 6.8% increase in specificity (from .73 to .78), 5.8% increase in sensitivity (from .69 to .73), 15.4% increase in PPV (from .39 to .45), and 2.2% increase in NPV (from .90 to .92). Furthermore, the test results showed that the performance of the proposed model could be largely enhanced through physical fitness tests in 3 evaluation indices: 10.6% increase in accuracy (from .682 to .754), 16.4% increase in AUC (from .67 to .78), and 30.0% increase in specificity (from .60 to .78).

**Conclusions:**

The proposed model presents a promising method to classify people with knee OA when compared with other classification models and the traditional BN model. It could be implemented in clinical practice as a prescreening tool for knee OA, which would not only improve the quality of health care for elderly people but also reduce overall medical costs.

## Introduction

### Background

Knee osteoarthritis (OA) is a progressive and irreversible condition affecting more than 250 million people around the world [1,2]. Early identification of knee OA is important, as it can improve treatment outcomes and reduce medical costs [3]. There are 2 traditional identification methods: imaging-based metrics (eg, x-rays and magnetic resonance imaging [MRI]) and patient-reported metrics (eg, pain). However, imaging-based metrics have some limitations: x-rays are not suitable for pregnant women, MRI is expensive, and both of them lack portability [4]. Meanwhile, patient-reported metrics are subjective and inconsistent [5]. To overcome these limitations, several studies have attempted to develop classification or prediction models to identify knee OA. The key elements of these models are algorithms and dataset attributes. Commonly used algorithms include logistic regression (LR) [2,6,7] and artificial neural network [8]. Commonly used dataset attributes include biometric characteristics [2,6,7,9,10] (eg, age, gender, and body mass index [BMI]) and other medical information [2,6,7] (eg, knee pain, occupational risks, and medical tests scores). The identification accuracy of these models is around 70%. However, there are 2 main issues surrounding these models [11]. First, data processing (reasoning and expression) is hard for both therapists and patients to understand; for example, as data processing within artificial neural networks is encapsulated and abstract, the study of their structures contributes little to their results (eg, there is no simple link between the network topology and the results). Second, the dataset attributes in some studies are too complicated; for example, 1 dataset [10] of 186 attributes contained variables from radiographs (eg, medial alignment angle), as well as biochemical markers from serum and urine (eg, fibulin 3-1), making them difficult and costly to collect.

### Research Motivations

Bayesian network (BN), in contrast, has the advantage of being applicable in classification or prediction models. Because its procedures of reasoning and expression can be easily understood and accepted by both therapists and patients, unlike the *black box* of other traditional algorithms, it is also able to present uncertainties and causalities, which are both important in the medical domain [12]. Several studies have examined the performance of BN by developing mathematical models for diagnosing different diseases, including breast cancer [13], lung cancer [14], and Alzheimer disease [12,15]. These experimental results showed that all models of these disease diagnoses provided accuracy of at least 80%, and their network structures could be easily understood. To date, only 1 study has been conducted using the BN model for the identification of knee OA [5]. Although the model is helpful in identifying the relationship between different risk factors, the practical clinical implications are minimal because the used radiographic data (eg, joint space narrowing) can be directly used to diagnose knee OA even without the model.

On the other hand, researchers reported that the results of simple physical fitness tests could provide useful information to help assess bodily functions or diseases [16-18]. On the basis of the report by Dobson [19], several physical fitness indices have been used to identify knee OA, such as the Timed Up and Go (TUG) test and the 6-min walk test (6MWT). These physical fitness tests have been applied in clinical practice [20,21]. Compared with other biomarkers, physical fitness scores are easily measured using low-cost equipment, making them suitable for community health centers.

### Research Purpose

The main purpose of this research was to propose a BN-based classification model for classifying people with knee OA. Specifically, the proposed BN will be modeled via a combination of expert knowledge and data-oriented modeling. Its network structure will be manually constructed based on a systematic review of literature and experts’ opinions, and automatically retrained by the BS learning algorithm [22]. Its network parameters will be learned by the expectation-maximization (EM) algorithm [23], and its dataset attributes will include backgrounds (5 attributes, subjects’ basic characteristics), the target disease, and predictors (13 attributes, physical fitness tests scores). The proposed model from this research could be implemented in clinical practice as a prescreening tool for knee OA, which could promote proactive knee OA prevention. The rest of the paper is organized as follows: Methods section details the dataset attributes used for training and validation, and the procedures for building the BN model; Results section presents the experimental result, which is discussed in the Discussion section, followed by the conclusive remarks in Conclusions.

## Methods

### Subjects and Data Measurement

This research used a dataset from a previous study (titled *The effectiveness of a combined exercise intervention on physical fitness factors related to falls in community-dwelling older adults* [24]), which was approved by Ethics Advisory Committee of Shanghai University of Sport. All participants gave their written informed consent before study. Subjects (aged between 60 and 80 years) were given an orientation (eg, study objectives, risks and benefits, and data collection procedures) and were asked to sign a consent form. The following basic characteristics were then collected from each subject through a questionnaire and a basic measurement: disease condition, gender, age, level of education, height, weight, waist girth, and hip girth. A total of 6 physical fitness tests were conducted after the basic characteristics collection: the single-leg stance balance (SLSB) test, body reaction time (BRT) test, modified sit and reach (MSR) test, leg extension power (LEP) test, TUG test, and Star Excursion Balance Test (SEBT). These tests provide different indices of physical fitness and/or activities of daily living for participants (Table 1). Their reliability [25-30] and predictive validity for knee OA have been verified [24,31-34]. The duration of the whole experiment for each subject was approximately 1 hour, and the detailed measurement of these 6 physical fitness tests has been presented in Multimedia Appendix 1.

**Table 1.** The measurements of 6 physical fitness tests.

Test	Measurement	Unit	ICC^a^
Single-leg stance balance test	Duration of body balance	Seconds	.994 [25]
Body reaction time test	Time of body reaction	Seconds	.915 [26]
Modified sit and reach test	Distance reached by the tip of the fingers	Centimeters	.980 [27]
Leg extension power test	Extension power of the leg muscles	Watts	.900 [28]
Timed Up and Go test	Time taken to finish the test (go and come back)	Seconds	.990 [29]
Star Excursion Balance Test	Distance between both feet (8 directions)	Centimeters	.990 [30]

^a^ICC: intraclass correlation coefficient.

### Data Analysis and Preprocessing

Before constructing the BN model, the collected data are preprocessed: some original attributes of background information are merged with new attributes, which are more sensitive to knee OA. According to the studies by Zhang [2] and Gandhi [35], BMI and waist-to-hip ratio (WHR) are common risk factors for knee OA, with their predictive validity being well verified. Therefore, in this research, BMI is used instead of height and weight, and WHR is used instead of waist girth and hip girth. Furthermore, Creamer [36] reported that education level is related to knee OA, as it influences the self-reported pain severity of knee OA. Thus, 5 basic characteristics (gender, age, BMI, WHR, and education level) of participants are determined.

According to biostatistics literature [37], data will lose its measure of confidence if its missing value ratio is greater than 30%. Therefore, for our research, some instances were removed from the dataset if they had more than 6 missing attributes (6 of 18). These missing attributes are normally caused by time conflicts and failures in the tests. As a result, a total of 131 instances were used as the primary dataset. The missing values of the primary dataset (11 of 2489) were then imputed using a filter commonly used in data mining classification techniques. The filter named *ReplaceMissingValues* then scanned all the values and replaced the missing values with mean values [38,39]. The demographic characteristics of the primary dataset have been presented in Table 2. Furthermore, according to recent literature [12,14], an imbalanced dataset will cause a skewed classification of the predicting target. In other words, the classification model will have high accuracy for the majority class but low accuracy for the minority class. As for our research, the states in the targeted disease are imbalanced: 40.5% positive cases and 59.5% negative cases (Table 2). To balance the dataset, the synthetic minority oversampling technique method was used. This method allows oversampling of the positive cases with little change in the characteristic of the primary dataset [40], and it has been used by many researchers to process imbalanced datasets [12,41]. Finally, a total of 157 instances were adopted in the final dataset, which contained 50.3% positive cases and 49.7% negative cases. The demographic characteristics of the final dataset are presented in Table 2.

**Figure 1:**
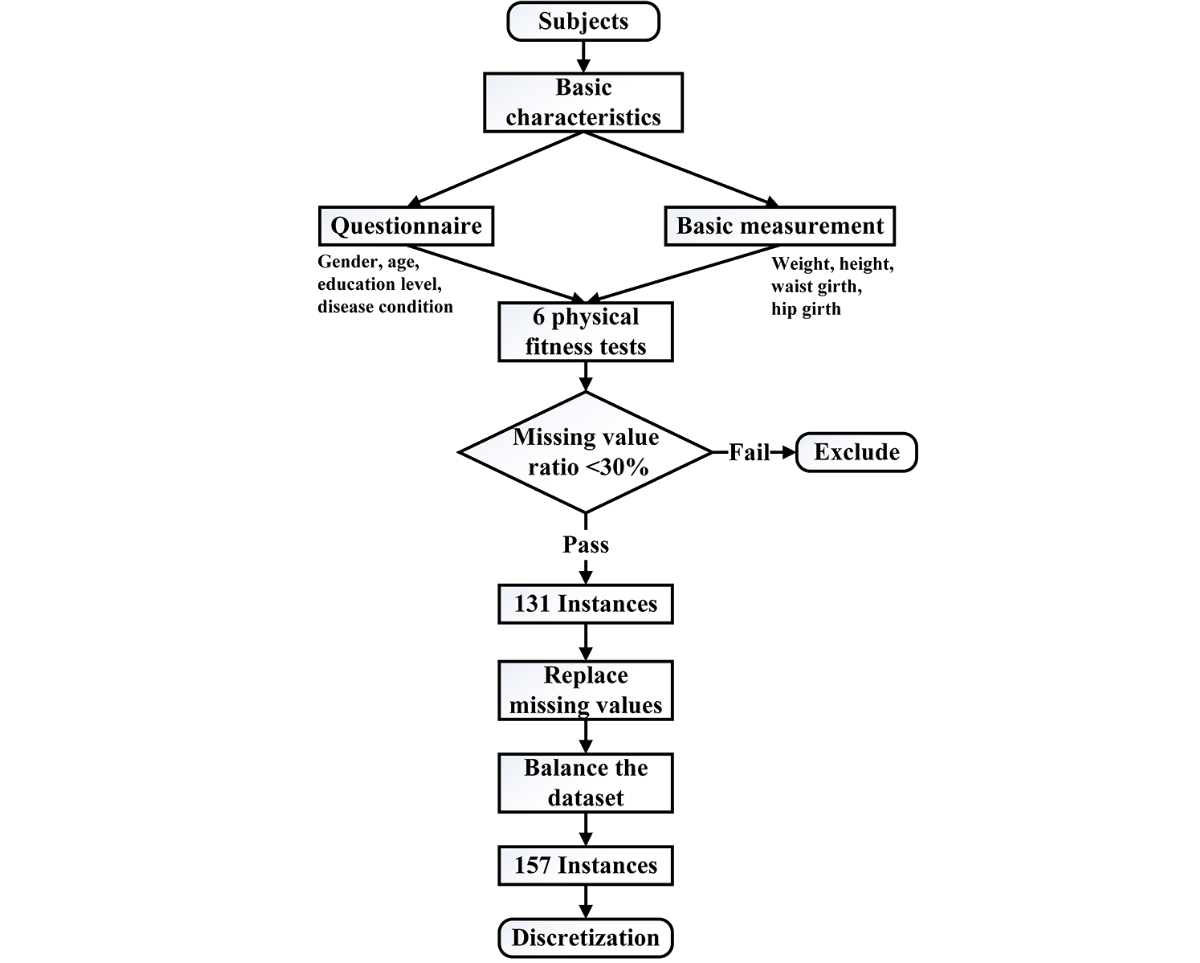
Flowchart of the data collection and preprocessing steps.

There are 2 types of variables, which can be handled by the BN model: continuous variables and discrete variables. Normally, most BN models will handle discrete variables [12,14,42]. In this research, we also focused on discrete variables for 3 reasons: (1) the results of our model are discrete; (2) the influence of abnormal values could be avoided, thus making the model more robust; and (3) discrete variables provide better interactions with users, as evidence could be easily selected from a set (eg, the user could select *good, moderate, or bad* from the test results). A simple k-means algorithm was used to cluster and estimate the cutting point of each continuous attribute. All the filters and algorithms are available in WEKA 3.6 (The University of Waikato, ‎Hamilton‎, ‎Waikato‎, New Zealand), a popular machine learning software [43]. The discretization results are presented in Table 3, and the procedure for data collection and preprocessing is presented in Figure 1.

**Table 2.** The demographic characteristics of the subjects.

Attribute		Primary (N=131)	Final (N=157)
**Gender,** **n** **(%)**			
	Male	45 (34.4)	54 (34.4)
	Female	86 (65.6)	103 (65.6)
Age (years), mean (SD)		70.37 (5.70)	70.31 (5.56)
Body mass index (kg/m^2^), mean (SD)		25.25 (3.89)	25.31 (3.74)
Waist-to-hip ratio, mean (SD)		0.91 (0.08)	0.92 (0.08)
**Education,** **n** **(%)**			
	Junior and below	38 (29.0)	47 (29.9)
	Junior high	41 (31.3)	61 (38.9)
	Senior high and above	44 (33.6)	49 (31.2)
	Missing	8 (6.1)	—^a^
**Osteoarthritis,** **n** **(%)**			
	Negative	78 (59.5)	78 (49.7)
	Positive	53 (40.5)	79 (50.3)
**Physical fitness test and unit, mean (SD)**			
	Single-leg stance balance test (eyes open, s)	72.77 (81.84)	70.30 (79.93)
	Body reaction time test (s)	0.63 (0.17)	0.64 (0.17)
	Modified sit and reach test (3 missing, cm)	24.47 (9.47)	24.55 (9.07)
	Leg extension power test (w)	287.41 (258.30)	289.62 (278.33)
	Timed Up and Go test (s)	8.85 (2.02)	8.91 (2.02)
	Anterior Star Excursion Balance Test^b^	0.77 (0.11)	0.76 (0.11)
	Anterolateral Star Excursion Balance Test^b^	0.83 (0.10)	0.83 (0.10)
	Lateral Star Excursion Balance Test^b^	0.81 (0.13)	0.81 (0.13)
	Posterolateral Star Excursion Balance Test^b^	0.77 (0.15)	0.77 (0.15)
	Posterior Star Excursion Balance Test^b^	0.67 (0.18)	0.66 (0.17)
	Posteromedial Star Excursion Balance Test^b^	0.61 (0.17)	0.61 (0.17)
	Medial Star Excursion Balance Test^b^	0.50 (0.16)	0.50 (0.15)
	Anteromedial Star Excursion Balance Test^b^	0.68 (0.11)	0.69 (0.11)

^a^Data not available.

^b^The measured value for the Star Excursion Balance Test has been normalized (without unit).

**Table 3.** The discretization results of the final dataset.

Level and attribute		States
**Background**	
	Gender	1: male; 2: female
	Age (years)	1: [0 to 70]; 2: (70 to infinity)
	Body mass index (kg/m^2^)	1: [0 to 25); 2: [25 to infinity)
	Waist-to-hip ratio	1: [0 to 0.91]; 2: (0.91 to infinity)
	Education	1: junior and below; 2: junior high; 3: senior high and above
**Disease**		
	Osteoarthritis	1: negative; 2: positive
**Predictor**		
	Single-leg stance balance test (s)	1: [0 to 73.6]; 2: (73.6 to infinity)
	Body reaction time test (s)	1: [0 to 0.63]; 2: (0.63 to infinity)
	Modified sit and reach test (cm)	1: [0 to 24.3]; 2: (24.3 to infinity)
	Leg extension power test (w)	1: [0 to 281]; 2: (281 to infinity)
	Timed Up and Go test (s)	1: [0 to 8.9]; 2: (8.9 to infinity)
	Anterior Star Excursion Balance Test	1: [0 to 0.763]; 2: (0.763 to 2.00)^a^
	Anterolateral Star Excursion Balance Test	1: [0 to 0.833]; 2: (0.833 to 2.00)^a^
	Lateral Star Excursion Balance Test	1: [0 to 0.812]; 2: (0.812 to 2.00)^a^
	Posterolateral Star Excursion Balance Test	1: [0 to 0.749]; 2: (0.749 to 2.00)^a^
	Posterior Star Excursion Balance Test	1: [0 to 0.658]; 2: (0.658 to 2.00)^a^
	Posteromedial Star Excursion Balance Test	1: [0 to 0.607]; 2: (0.607 to 2.00)^a^
	Medial Star Excursion Balance Test	1: [0 to 0.490]; 2: (0.490 to 2.00)^a^
	Anteromedial Star Excursion Balance Test	1: [0 to 0.682]; 2: (0.682 to 2.00)^a^

^a^The measured value for the Star Excursion Balance Test has been normalized.

### Bayesian Network Concept and Modeling

**Figure 2:**
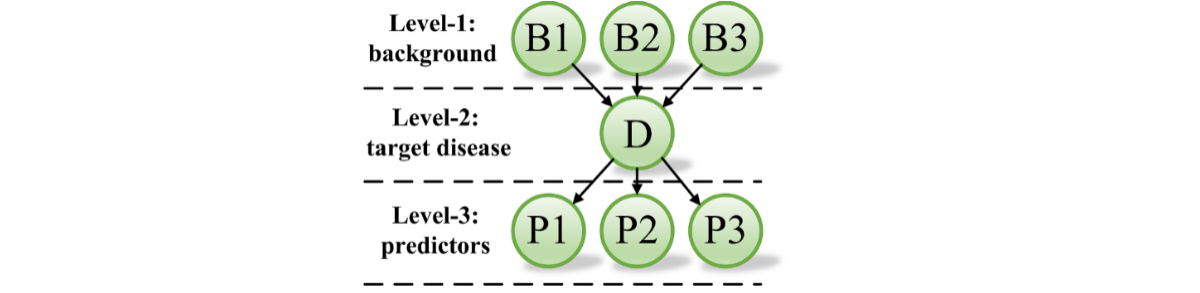
Three-level Bayesian network model in the medical domain.

The BN is a probability graphical model, which describes a set of random variables and their conditional dependencies through a directed acyclic graph [44]. The key elements in building a BN are its structure and parameters. The structure contains nodes and their directed edges: each node expresses a variable of the BN, and each directed edge represents a direct dependency between each pair of nodes. The parameters (conditional probability tables) represent prior knowledge of each node, which can be obtained from experts or specialized learning algorithms. Once the structure and parameters are determined, the results (posterior probability distribution, eg, the percentages of knee OA and not knee OA) of query variables will be calculated by the inference engine each time a user inputs evidence. A simple example of a 3-level BN model, including the background level, target disease level, and predictor level, in the medical domain is shown in Figure 2. The background level contains subjects’ basic information such as gender, age, and education; the target disease level shows the predicted disease; and the predictor level presents the predictors, which include signs, symptoms, and the test results. The basic principle of conditional probability is based on Bayes’ theorem:

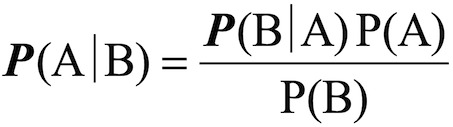



where A and B are events, and P(B)≠0 [42]. A basic 3-level BN model in the medical domain for the diagnosis of tuberculosis has been attached as Multimedia Appendix 2.

**Figure 3:**
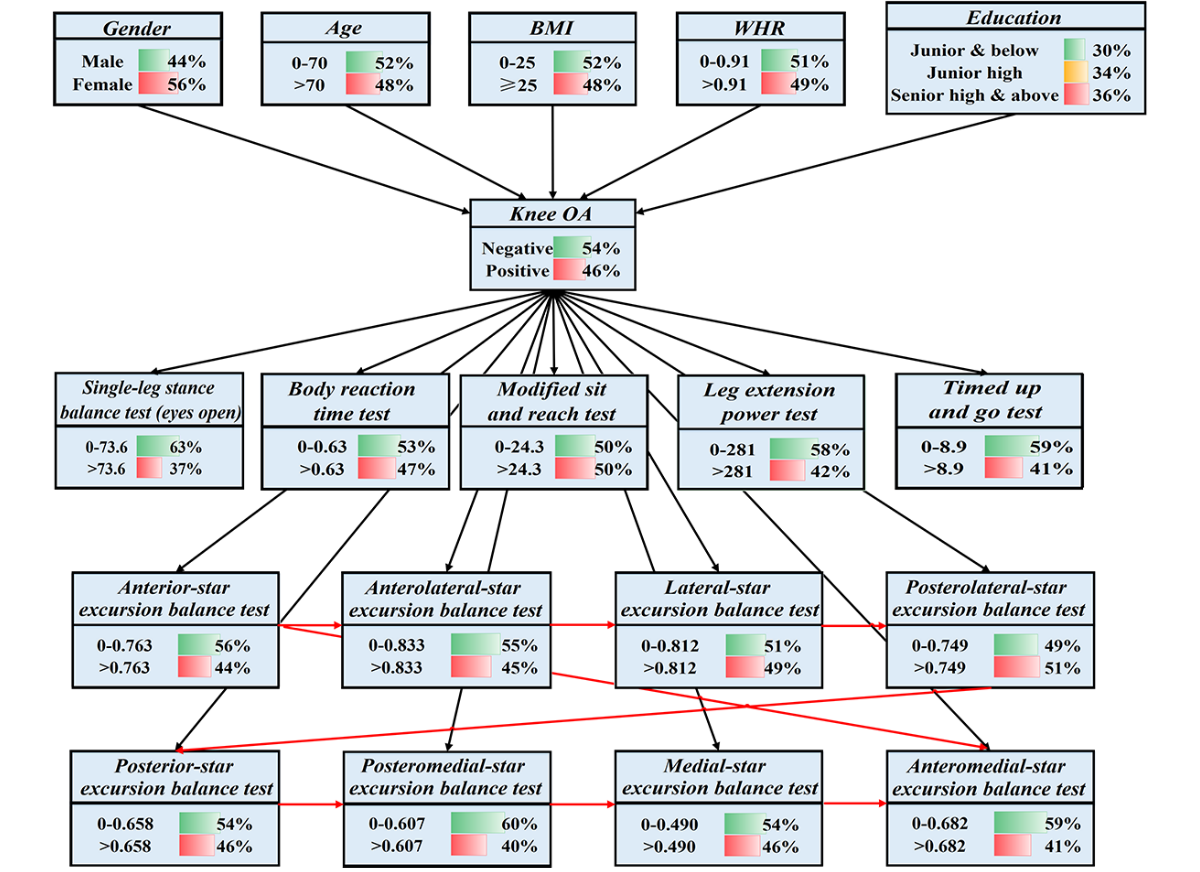
The semihandcrafted Bayesian network model. BMI: body mass index; WHR: waist-to-hip ratio.

**Figure 4:**
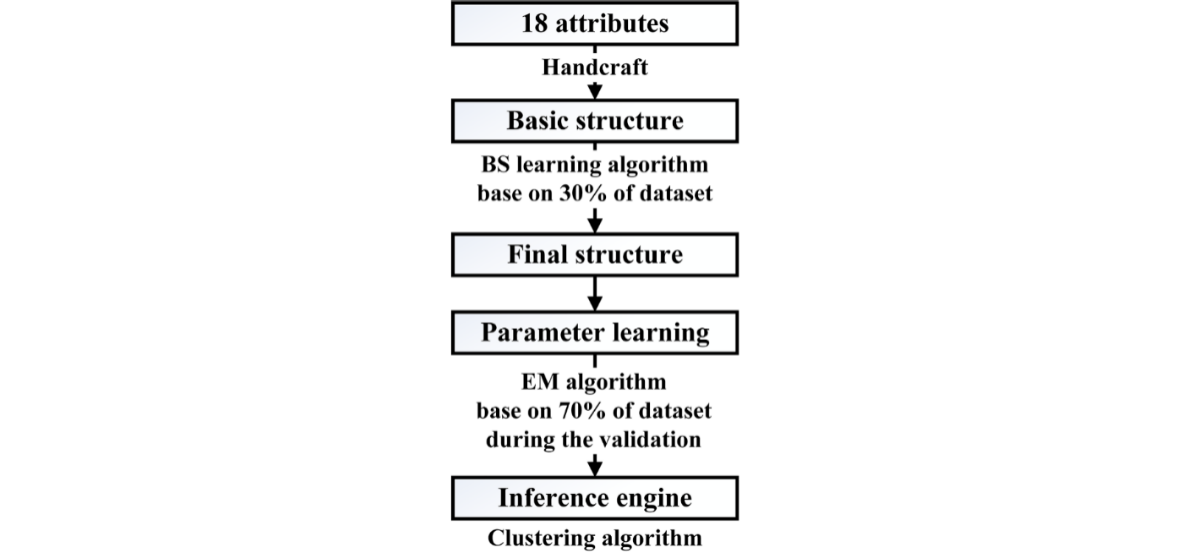
The procedure for building the semihandcrafted Bayesian network model. BS: Bayesian Search; EM: Expectation-Maximization.

As discussed in the section previously, BN modeling mainly contains 2 tasks: structure learning and parameter learning. During structure learning, we develop a semihandcrafted network structure. The basic structure (Figure 3, the black lines) is constructed according to related knee OA literature [2,3,5,45] and is examined by domain experts. Specifically, 5 basic characteristics (gender, age, BMI, WHR, and education) of participants are set as the background level, knee OA is set as the target disease level, and 6 physical fitness tests (SLSB, BRT, MSR, LEP, TUG tests, and SEBT [it has 8 directions]) are set as the predictor level. As mentioned previously, the selected basic characteristics are commonly used risk factors for knee OA [2,35,36], and the selected physical fitness tests have been verified to be effective in predicting knee OA as well [24,31-34]. Moreover, the basic structure is retrained by the BS learning algorithm based on 30% of the final dataset to get the improved structure, and some hidden relationships between attributes are found as well (Figure 3, the red lines, will be discussed later). The used BS learning algorithm adopts the classification accuracy (k-fold cross-validation method, *k*=5) as the scoring function in search for the optimal structure [46]. Meanwhile, the EM algorithm is used for parameter learning based on the rest of the final dataset during validation. This algorithm has the ability to learn parameters of a given BN structure from the dataset that contains missing values [23]. Furthermore, the clustering algorithm is used as the inference engine because our BN model is simple (a total of 18 attributes). The whole procedure for building the proposed semihandcrafted BN (SHBN) model has been shown in Figure 4. In this research, the BN toolbox in Matlab 2016b (MathWorks Inc., Natick, MA, USA) was used to determine the structure and parameters, and GeNIe 2.2 (BayesFusion LLC, Pittsburgh, PA, USA) was used as the interface engine to allow users to interact with the BN model and view the results. It should be noted that we kept both the basic handcrafted BN (HBN) and SHBN models to explore whether the performance of the traditional BN model can be improved by advanced learning algorithms (in the aspect of structure).

## Results

### Model Evaluation Criteria

The proposed SHBN model is evaluated against 2 criteria: the classification performance and the robustness. The classification performance (eg, classification accuracy and area under the curve [AUC]) evaluates how well the SHBN model differentiates between 2 states: positive or negative of having knee OA. The robustness (eg, specificity and sensitivity) evaluates the SHBN model’s ability to handle uncertainty in the output, which could be affected by the evidence from the input. The specificity here can be named the true negative rate. It can reflect the proportion of healthy subjects who are correctly identified as not having the knee OA. The sensitivity here can be named the true positive rate. It can reflect the proportion of sick subjects who are correctly identified as having the knee OA. To verify the classification performance and robustness of the SHBN model, 6 well-known classification models are selected to make comparisons [11]: decision tree (DT), discriminant analysis, LR, support vector machine, k-nearest neighbor (KNN), and ensemble method (discriminant subspaces-based ensemble method). These classification models have been used by many researchers to identify or classify people with knee OA [2,47,48], and the used Ensemble method is known for processing binary classification [49]. The detailed information (kernel and parameter) of these classification models can be seen in Table 4 and is also available in the Classification Learner App, MATLAB. Furthermore, to explore whether the physical fitness tests could improve the performance of the SHBN model, a test was conducted based only on the subjects’ basic characteristics, including gender, age, education level, BMI, and WHR. The k-fold cross-validation method was used for all models based on 70% of the final dataset (*k*=5, and the other 30% was specifically used to train the BN structure as described above). The experimental results have been shown in Table 5. On the other hand, knee OA is a condition with increased prevalence. It is necessary to compare the positive predictive value (PPV) and negative predictive value (NPV) of each model according to an apriority probability (the prevalence of knee OA) of 1%, 10%, and 20% [50]. The PPV here means the ability of a model to detect the presence of disease. The NPV here means the ability of a model to detect the absence of disease. PPV and NPV are of high interest for clinical applications; the experimental results are presented in Tables 6 and 7.

### Experimental Results

A total of 249 elderly people aged between 60 and 80 years, living in the Kongjiang community (Shanghai), were recruited from April to September 2007. The ethical approval was obtained from the Ethics Advisory Committee of Shanghai University of Sport. After data preprocessing, a total of 157 instances were adopted as the dataset, which included backgrounds (5 attributes, the basic characteristics of subjects), the target disease (namely the knee OA), and predictors (13 attributes, the scores of physical fitness tests). Table 5 showed that the proposed SHBN model presented a promising result when compared with other classification models, and the scores for all evaluation indices were higher (or equal) than the mean scores. Specifically, based on the criteria of classification performance, (1) for classification accuracy, the Ensemble model received the highest score (.773) followed by the SHBN model (.754) and (2) for the AUC, the Ensemble and LR models received the highest score (.81), whereas the SHBN model (.78) ranked third with little difference with the other scores. On the basis of the criteria of robustness, (1) for specificity, the Ensemble, LR, and SHBN models received the highest score (.78) and (2) for sensitivity, the DT and KNN models received the highest score (.78), whereas the SHBN model (.73) ranked fourth. It should be noted that the HBN model showed a moderate result, and no evaluation indices were better than the mean scores.

**Table 4.** Detailed information of well-known classification models.

Model and kernel			Parameter
**Decision tree**			
	Medium tree		Maximum number of splits: 20; Split criterion: Gini’s diversity index
**Discriminant analysis**		
	Quadratic discriminant		Regularization: diagonal covariance
**Logistic regression**		
	Fitlm function		Chi-square statistic versus constant model: 65.1
**Support vector machine**		
	Gaussian SVM^a^		Kernel scale: 2.2; Box constraint level: 1; Multiclass method: 1 versus 1
**K-nearest neighbor**		
	Medium KNN^b^		Number of neighbors: 10; Distance metric: Euclidean; Distance weight: equal
**Ensemble method**		
	Subspace with discriminant learner		Number of learner: 30; Subspace dimension: 3

^a^SVM: support vector machine.

^b^KNN: k-nearest neighbor.

**Table 5.** The performance of models with different criteria.

Model	Accuracy	Area under the curve	Specificity	Sensitivity
Semihandcrafted Bayesian network	.754	.78	.78	.73
Handcrafted Bayesian network	.709	.75	.73	.69
Decision tree	.736	.77	.69	.78
Discriminant analysis	.709	.75	.73	.69
Logistic regression	.736	.81	.78	.69
Support vector machine	.709	.77	.73	.69
K-nearest neighbor	.727	.78	.67	.78
Ensemble method	.773	.81	.78	.76
Mean score^a^	.732	.78	.73	.73
Semihandcrafted Bayesian network^b^	.682	.67	.60	.76
Logistic regression^b^	.709	.74	.73	.69

^a^The mean score includes the results of the DT, DA, LR, SVM, KNN, and Ensemble method models.

^b^These results are based only on the subjects’ basic characteristics, without the scores of physical fitness tests.

**Table 6.** The positive predictive values of models in different conditions.

Model	The apriority probability		
	1%	10%	20%
Semihandcrafted Bayesian network	.03	.27	.45
Handcrafted Bayesian network	.03	.22	.39
Decision tree	.02	.22	.39
Discriminant analysis	.03	.22	.39
Logistic regression	.03	.26	.44
Support vector machine	.03	.22	.39
K-nearest neighbor	.02	.21	.37
Ensemble method	.03	.28	.46

**Table 7.** The negative predictive values of models in different conditions.

Model	The apriority probability		
	1%	10%	20%
Semihandcrafted Bayesian network	1.00	.96	.92
Handcrafted Bayesian network	1.00	.95	.90
Decision tree	1.00	.97	.93
Discriminant analysis	1.00	.95	.90
Logistic regression	1.00	.96	.91
Support vector machine	1.00	.95	.90
K-nearest neighbor	1.00	.96	.92
Ensemble method	1.00	.97	.93

Furthermore, the results of the test showed that the physical fitness tests improved the performance of the classification models, especially for our SHBN model. Specifically, without the attributes of physical fitness tests, the identification accuracy of the SHBN model decreased from .754 to .682, the AUC score decreased from .78 to .67, the specificity score decreased from .78 to .60, but the sensitivity score increased from .73 to .76. The result from the LR model followed a similar trend: the identification accuracy decreased from .736 to .709, the AUC score decreased from .81 to .74, and the specificity score decreased from .78 to .73. The sensitivity score stayed the same (.69). In addition, the results of PPV (Table 6) showed that the Ensemble model received the highest scores in all conditions followed by the SHBN model. The results of NPV (Table 7) presented a similar trend that the Ensemble and DT models received the highest scores in all conditions, whereas the SHBN model received moderate scores in all conditions. It is worth noting that the HBN model ranked fourth for PPV, whereas it ranked last for NPV.

## Discussion

### Principal Findings

The main findings of this research are as follows: (1) the proposed SHBN model presents satisfactory performance to classify people with knee OA in all evaluation indices (accuracy, AUC, specificity, sensitivity, PPV, and NPV); and (2) the proposed SHBN model presents a significant improvement in all evaluation indices when compared with the traditional BN model.

The performance of the SHBN model have been discussed: (1) comparisons with other well-known classification models; (2) comparisons with other BN-based models; and (3) comparisons with traditional HBN model.

First, the performance of each model has been shown in Table 5. Specifically, the SHBN model provided the best specificity (.78), which was the same as the LR and Ensemble models, whereas the highest classification accuracy was achieved by the Ensemble model (.773), the highest AUC was achieved by the LR and Ensemble models (.81), and the best sensitivity was achieved by the DT and KNN models (.78). These results are similar to the research of Seixas [12], in which the BN model did not show the best result as well. The possible reason for this could be that the Ensemble model combines multiple models (eg, subspace analysis and discriminant learner), which produces better performance than a single model [51]. Meanwhile, the BN model has its own shortcomings: some complicated scoring functions require reliable prior knowledge to find a structure that is closer to the realistic model [11]. In this research, the final structure was trained based on the 30% of the dataset, which could not cover all instances. The reason for not using the whole dataset in the learning of structure and parameter is that it might cause overfitting by using the same dataset to do the cross-validation [52]. In fact, during structure learning, the k-fold cross-validation method was used as the scoring function in searching for the optimal structure. In other words, all the results were tested by the cross-validation method. In addition, in terms of PPV and NPV, the SHBN model showed a promising result. Specifically, for PPV (Table 6), the SHBN model received .03, .27, and .45 with the apriority probability (the prevalence of knee OA) of 1%, 10%, and 20%, respectively. For NPV (Table 7), the SHBN model received 1.00, .96, and .92 with the same trend of the apriority probability. These results are slightly better than the results reported by Peat [53]: .44 for PPV and .72 for NPV with the DT method at the prevalence of 30%. In addition, data from Tables 6 and 7 indicated a trend that PPV and NPV vary with increased prevalence for all models. In other words, in a dataset with higher prevalence of knee OA, PPV increased and NPV decreased, which is supported by Peat [53] as well.

Second, as discussed in the Introduction section, the BN can provide above 80% accuracy for identifying other diseases. Although knee OA is different from these diseases, 3 possible reasons for the imperfect identification accuracy of our SHBN model were hypothesized. (1) The used dataset was not complicated (only contained 18 attributes), and these attributes came from general information including the basic characteristics of subjects and simple physical fitness scores, rather than special radiographic data such as joint space narrowing. The main reason for using such dataset is to achieve one of the purposes of this research, that is, to develop a classification model for knee OA, which could be easily performed by normal operators, and the used dataset attributes could be collected by cheap and portable equipment, no matter in community health centers or rural hospitals. Therefore, special radiographic data could not be included despite being able to largely improve the performance of the proposed model. (2) The used dataset was not large (N=157), and there is no doubt that the identification accuracy would be enhanced if a larger dataset is used instead, for example, Wang [14] adopted 4555 instances and achieved .82 accuracy. (3) The skewed dataset might have an impact on the performance, which is suggested by Watt [5], for example, the females covered 66% of total instances (Table 2). However, because gender is an attribute rather than the target node, it should not be balanced.

Third, the performance of the traditional HBN model across the different evaluation indices was lower than the mean score and of other classification models (Table 5). The results of NPV were also worse than those of other classification models (Table 7). Possible reasons could be similar to that of the SHBN model in which the used attributes were not complicated enough and the dataset was not large enough. However, the SHBN model presented a significant improvement in all evaluation indices when compared with the HBN model: the percent gains for the identification accuracy, the AUC score, the specificity score, the sensitivity score, the PPV, and the NPV were 6.3% (from .709 to .754), 4.0% (from .75 to .78), 6.8% (from .73 to .78), 5.8% (from .69 to .73), 15.4% (from .39 to .45, at the prevalence of 20%), and 2.2% (from .90 to .92, at the prevalence of 20%), respectively. A possible reason for this has been explained by Watt [5], where the subjectivity of the handcrafted network structure could bring bias into the modeled BN relations. Due to this, alternative method should be used to automatically suggest the network structure from the dataset. Moreover, Seixas [12] reported a similar finding in which the BN model discovered from a dataset revealed a slight improvement in some evaluation indices. In that research, the structure of the model was automatically built by the learning algorithm but was problematic because it treated the symptoms as risk factors of the disease, which are incorrect for the diagnosis criteria. Therefore, our research combines the traditional handcrafted approach and the learning algorithm to address this problem (which is why the structure is named *semihandcrafted*). The final structure of the SHBN can be seen in Figure 3, in which several hidden relationships (red lines) between the 8 directions of SEBT are discovered. It is acceptable that there are correlations between these directions because they belong to the same physical fitness test. In other words, if the result of *anterior* direction is *high*, there is a great probability of other directions’ results to be *high*. Meanwhile, no correlation has been found among other physical fitness tests because all of them are independent of each other. It should be noted that if the used dataset is to be changed, the discovered structure may be changed as well. However, in this research, we want to show the possibility that the traditional HBN model can be improved, which has been well verified by the experimental results in all evaluation indices. On the other hand, in fact, no structure can be treated as a *one-for-all* structure; the practical BN model should be adjusted to meet different requirements of users.

Although the performance of the SHBN model is not the best for all evaluation indices, it still has some advantages in the identification of knee OA. (1) The proposed model has the ability to graphically present the procedures of reasoning and expression, which can help therapists and patients to understand the diagnosis criteria. (2) Due to the used 3-level structure, the proposed model can provide a clearer human-oriented diagram than that of traditional BN models [54]. (3) The proposed model is robust when facing missing values and will create the best possible result with whatever evidence is inputted (dataset with missing values, unfortunately, is the typical case in the medical domain). For example, if 1 subject cannot finish the MSR test and TUG test, the therapist can still use the remaining 16 attributes to identify the knee OA (example for predicting knee OA with missing values has been attached as Multimedia Appendix 3). In addition, the effectiveness of the physical fitness tests is confirmed by the results. Table 5 showed that the identification accuracy of the SHBN model increased from .682 to .754 (percent gain: 10.6%), which was similar for the AUC score (from .67 to .78, percent gain: 16.4%) and specificity score (from .60 to .78, percent gain: 30.0%). The performance of the LR model was also improved but was not very obvious when compared with the SHBN model: the percent gains for the identification accuracy, the AUC score, and the specificity score were 3.8% (from .709 to .736), 9.5% (from .74 to .81), and 6.8% (from .73 to .78), respectively. A similar result was reported by Zhang [2], in which risk prediction models were developed for knee OA based on LR model, and some basic biometric characteristics (age, gender, BMI, and so on) were used as the predictors. Around .75 of the AUC were calculated by these risk prediction models, which is almost the same result as the LR model (.74) in our test using the attributes of subjects’ basic characteristics.

In general, the performance of the proposed SHBN model is promising and satisfactory when compared with other well-known models and other BN models, which reveals a good identification result. Meanwhile, the SHBN model shows a significant improvement in all evaluation indices when compared with the HBN model, which confirms that the reliability and validity of the traditional HBN model can be improved by advanced learning algorithms.

### Potential Clinical Significance and Future Work

As discussed in the Introduction section, early identification of knee OA is important to support the timely adjustment of appropriate clinical interventions. In this research, several commonly used basic characteristics of subjects were adopted as inputs for our model to overcome issues that hinder the identification of knee OA, for example, the frequent use of expensive diagnosis tools and special equipment. Meanwhile, to improve the performance of our model, the scores of 6 physical fitness tests were used as the inputs as well. These 6 physical fitness tests can be easily performed in community health centers, and the required equipment is cheap and portable. There are also some advantages in using the BN model in the medical domain [55] such as adaptability and strong robustness against missing values. Regarding adaptability, the BN model can be started with small and limited domain knowledge and then further extended (or simplified) by inputting new knowledge to suit different requirements. In practice, therapists can collect the up-to-date knowledge of each patient, and the probabilities in the BN model will be adjusted automatically. Regarding strong robustness against missing values, as discussed in the previous section, the BN model does not require complete knowledge of the instance and can use as much knowledge as available to do the predication.

In addition, 1 important clinical implication is that the proposed SHBN model can potentially be used as a cheap and portable prescreening tool to identify people with a high risk of knee OA. These identified people are then recommended to undergo further examination using traditional diagnosis tools (eg, x-rays and MRI). The successful identification and treatment of people with knee OA are beneficial for them and the government’s health care system because it can reduce long-term morbidity and overall medical costs [2]. Furthermore, the proposed SHBN model can also make the identification of knee OA easier, leading to the better quality of health care for elderly people.

### Limitations

This research has 2 limitations. First, the used dataset was not large (N=157), and it was not a random sample of the general population. Participants were all elderly people (aged between 60 and 80 years), and most of them resided in the Kongjiang community (Shanghai, China); therefore, the generalizability of the proposed model might be limited. Second, the disease condition of knee OA was self-reported, and the proposed SHBN model could only be treated as the classification model because the used dataset was extracted from the existing data. This warrants future work to overcome the limitations and improve the performance of the proposed model for processing new data by (1) recruiting more subjects with different age and locations to improve the generalizability of the proposed model and (2) including other physical fitness tests for other population groups.

### Conclusions

This paper proposes an SHBN model for the identification of knee OA. This model is based on a 3-level BN structure where background information, target disease, and predictors are linked using hierarchically structured random variables. A total of 157 instances with 18 attributes were used to constitute the subjects’ dataset, which included the basic characteristics of subjects and the scores of 6 physical fitness tests. The experimental results showed that the proposed SHBN model can provide a promising and satisfactory result in terms of classification performance (classification accuracy=.754 and AUC=.78), model’s robustness (specificity=.78 and sensitivity=.73), and predictive performance (PPV=.45 and NPV=.92 at the prevalence of 20%). In addition to this, the proposed SHBN model represents potential clinical significance because of its advantages, which can be used with appropriate prevention methods to reduce the risk of knee OA in elderly people and improve their quality of health care.

## Appendix

Multimedia Appendix 1 Detailed measurement for 6 physical fitness tests.

Multimedia Appendix 2 A basic 3-level Bayesian network model for the diagnosis of tuberculosis.

Multimedia Appendix 3 The diagnostic procedure for missing values.

## Abbreviations

AUC: area under the curve
BMI: body mass index
BN: Bayesian network
BRT: body reaction time
BS: Bayesian Search
DT: decision tree
EM: expectation-maximization
HBN: handcrafted BN
KNN: k-nearest neighbor
LEP: leg extension power
LR: logistic regression
MRI: magnetic resonance imaging
MSR: modified sit and reach
NPV: negative predictive value
OA: osteoarthritis
PPV: positive predictive value
SEBT: Star Excursion Balance Test
SHBN: semihandcrafted BN
SLSB: single-leg stance balance
TUG: Timed Up and Go test
WHR: waist-to-hip ratio
