# Supplementary material for: Identification of Knee Osteoarthritis Based on Bayesian Network: Pilot Study
Source: JMIR Med Inform. 2019 Jul 18;7(3):e13562. doi: 10.2196/13562 (PMC6670282; doi:10.2196/13562)
Supplement: Multimedia Appendix 1 [file medinform_v7i3e13562_app1.pdf]

Multimedia Appendix 1: Detailed measurement for 6 physical fitness tests.

The single-leg stance balance (SLSB) test is used to measure the body balance, which is performed by a balance performance monitor (Fitness Station, Combi Wellness, Japan). For safety purposes, subjects are advised to stand on one foot with the other off the monitor (eyes open and arms out). The time is recorded until balance is lost in such that the raised foot is lowered to the monitor [1]. Two trials are conducted on both the subjects' right and left foot, and the best score is recorded as the definitive measurement.

The body reaction time (BRT) test is carried out to measure their response to some sort of stimulus. The BRT test is conducted by a reaction time tester (Fitness Station, Combi Wellness, Japan), and subjects are advised to stand on the reaction time tester. When the light (the light is placed one meter away from the subjects) is on, the subjects jump as quickly as possible, and the reaction time is recorded. Five trials are performed for the BRT test and the best score is recorded as the definitive measurement.

The modified sit and reach (MSR) test is developed based on the traditional sit and reach (SR) test. This is modified so that the finger-to-box distance is measured to amend the proportional differences between the arm and leg lengths [2]. Several researches have reported that the score of the MSR test is not related to the arm and leg lengths, and therefore the MSR test can correctly reflect the hamstring flexibility, especially for old people [2, 3]. On the other hand, it is suggested that a lack of flexibility could be a risk factor for knee OA, that is, patients with keen OA show poor flexibility in both the affected and unaffected legs [4]. Subjects are advised to sit on the floor with their backs and heads against a wall. Their legs should be out straight ahead and knees flat against the floor, with a box placed flat against their feet. Subjects stretch their arms out towards the box to adjust the sliding ruler to the zero mark whilst keeping their backs and heads against the wall. Then subjects lean forward slowly as far as possible with their hands side by side, keeping their fingertips level with each other and their legs flat. They keep the full reach position for two seconds, and then record the results [5]. The MSR test is performed twice by an analog bend meter (TKK-5003, Takei Kiki Kogyo, Japan) and the mean score is treated as the definitive measurement.

The leg extension power (LEP) test is used to measure the extension power of the leg muscles. The LEP test is conducted by an isokinetic leg power system (Anaero Press 3500, Combi Wellness, Japan). Subjects are fastened by a seat belt to the system, and their feet are placed on a sliding plate with their knee angle at 90 degrees [6]. Subjects are advised to vigorously extend their legs five times, and the mean of the two highest scores is taken as the definitive measurement.

The timed up and go (TUG) test is used to measure the mobility and balance of old people. Subjects are advised to stand up from a chair, walk 3 meters, walk around a cone, and return to the chair as quickly as possible [7]. The TUG test is performed three times and the mean score is taken as the definitive measurement.

The star excursion balance test (SEBT) is also used to measure the mobility and balance for old people. Subjects are advised to stand on one leg in the center of a grid, and stretch their non-stance leg as far as possible in eight directions, including anterior, anterolateral, lateral, posterolateral, posterior, posteromedial, medial and anteromedial (the same as a compass rose) [7]. The SEBT is performed three times, and the mean score is normalized with the length of the non-stance leg of each subject. The normalized mean score is treated as the

definitive measurement.

Reference

1. Okuyama N, Yamaga T, Yoshihara A, Nohno K, Yoshitake Y, Kimura Y, et al. Influence of dental occlusion on physical fitness decline in a healthy Japanese elderly population. Arch Gerontol Geriatr 2011 Mar-Apr;52(2):172-6. [doi: 10.1016/j.archger.2010.03.011][Medline: 20378189]
2. Hoeger WW, Hopkins DR, Button S, Palmer TA. Comparing the sit and reach with the modified sit and reach in measuring flexibility in adolescents. Pediatr Exerc Sci 1990 May;2(2):156-62. [doi: 10.1123/pes.2.2.156]
3. Lemmink KA, Kemper HC, Greef MH, Rispens P, Stevens M. The validity of the sit-and-reach test and the modified sit-and-reach test in middle-aged to older men and women. Res Q Exerc Sport 2003 Sep;74(3):331-6. [doi: 10.1080/02701367.2003.10609099][Medline: 14510299]
4. Messier SP, Loeser RF, Hoover JL, Semble EL, Wise CM. Osteoarthritis of the knee: effects on gait, strength, and flexibility. Arch Phys Med Rehabil 1992 Jan;73(1):29-36. [Medline: 1729969]
5. Topend sports. 2018. Fitntess testing, Modified Sit and Reach Test; 2018. URL: https://www.topendsports.com/testing/tests/sit-and-reach-modified.htm [accessed 2019-01-31][WebCite Cache ID 75pJmTycY]
6. Zhang JG, Ohta T, Ishikawa-Takata K, Tabata I, Miyashita M. Effects of daily activity recorded by pedometer on peak oxygen consumption, ventilatory threshold and leg extension power in 30-to 69-year-old Japanese without exercise habit. Eur J Appl Physiol 2003 Sep;90(1-2):109-13. [doi: 10.1007/s00421-003-0860-0][Medline: 12827366]
7. Zhuang J, Huang L, Wu Y, Zhang Y. The effectiveness of a combined exercise intervention on physical fitness factors related to falls in community-dwelling older adults. Clin Interv Aging 2014;9:131-40. [doi: 10.2147/CIA.S56682][Medline: 24453483]
