# Supplementary material for: Identification of Knee Osteoarthritis Based on Bayesian Network: Pilot Study
Source: JMIR Med Inform. 2019 Jul 18;7(3):e13562. doi: 10.2196/13562 (PMC6670282; doi:10.2196/13562)
Supplement: Multimedia Appendix 2 [file medinform_v7i3e13562_app2.pdf]

Multimedia Appendix 2: A basic 3-level Bayesian network model for the diagnosis of tuberculosis

Figure A1 shows a basic three-level BN model in the medical domain for the diagnosis of Tuberculosis [1]. Firstly, a basic BN model (Figure A1 (a)) is set up according to the textbook. Secondly, the background information of a patient is acquired (Figure A1 (b), he visited Asia), and the result of Tuberculosis is updated according to Table A1 (the CPTs are only for the example). Meanwhile, the result of the 'XRay result' is calculated as:

$$\boldsymbol{P}(\mathrm{X}) = \boldsymbol{P}(\mathrm{X}|\mathrm{T})\boldsymbol{P}(\mathrm{T}) + \boldsymbol{P}(\mathrm{X}|\overline{\mathrm{T}})\boldsymbol{P}(\overline{\mathrm{T}}) = 0.4*0.4 + 0.3*0.6 = 0.34 \quad \text{(A1)}$$

where X is the abnormal of 'XRay result', T is the present of 'Tuberculosis' and $\overline{\mathrm{T}}$ (T bar) is the absent of 'Tuberculosis'. Thirdly, the patient is asked to perform an XRay test to get the predictor information. When the result of XRay test is inputted into the BN model (Figure A1 (c)), the probability of Tuberculosis is calculated as:

$$\boldsymbol{P}(\mathrm{T}|\mathrm{X}) = \frac{\boldsymbol{P}(\mathrm{X}|\mathrm{T})\mathrm{P}(\mathrm{T})}{\mathrm{P}(\mathrm{X})} = \frac{0.4*0.4}{0.34} \approx 0.47 \quad \text{(A2)}$$

The result shows that the probability of the Tuberculosis is increased a lot (from 30% to 40% to 47%, see Figure A1), which strongly suggests that further tests are needed for the participant, such as blood tests, lung tissue biopsies, and so on. These tests are not covered in this basic example, but they can be easily added to the predictor level (the same goes for the background level).

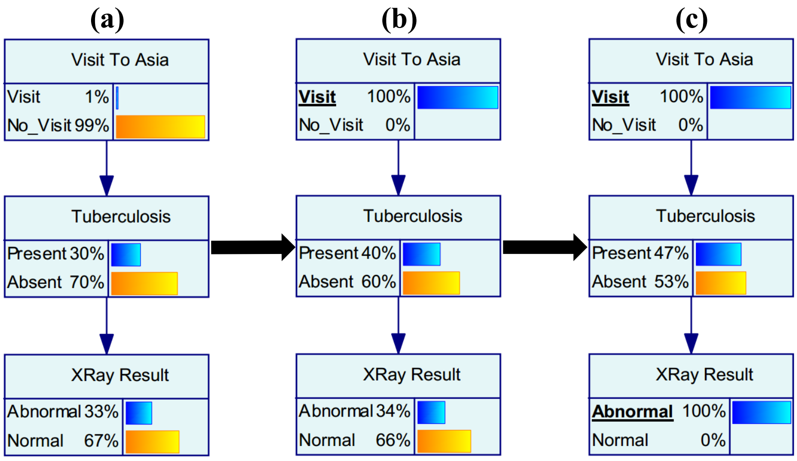

Figure A1. An example of the diagnostic procedure of a BN model: (a) the basic model, (b) the updated model with evidence from the background level, and (c) the updated model with evidence from the background level and predictor level.

Table A1. Conditional probability table for the given example.

Tuberculosis			XRay Result		
Visit To Asia	Visit	No_Visit	Tuberculosis	Present	Absent
Present	0.4	0.3	Abnormal	0.4	0.3
Absent	0.6	0.7	Normal	0.6	0.7

References

1. Norsys Software Corp. 2019. Netica tutorial: introduction to Bayes nets. URL: https://www.norsys.com/tutorials/netica/secA/tut_A1.htm [accessed 2019-01-31][WebCite Cache ID 75pGwqf1G]
