# Supplementary material for: Identification of Knee Osteoarthritis Based on Bayesian Network: Pilot Study
Source: JMIR Med Inform. 2019 Jul 18;7(3):e13562. doi: 10.2196/13562 (PMC6670282; doi:10.2196/13562)
Supplement: Multimedia Appendix 3 [file medinform_v7i3e13562_app3.pdf]

Multimedia Appendix 3: The diagnostic procedure for missing values

The diagnostic procedure of SHBN model with missing values has been shown in Figure A2. As introduced in the discussion section, the proposed SHBN model can process datasets with missing values. For example, after finishing the questionnaire and basic measurement, a subject's background information is put into the SHBN model: male, 75 years old, 24 for BMI, 0.80 for WHR, and junior high school education. He is then invited to do six physical fitness tests to get the required scores. However, the subject could not finish the 'Modified sit and reach test' and 'Timed up and go test', and so these two scores are not put into the SHBN model. Despite this, the results show that the subject has a 60% probability of being identified as not having knee OA, due to the good performance of the remaining tests.

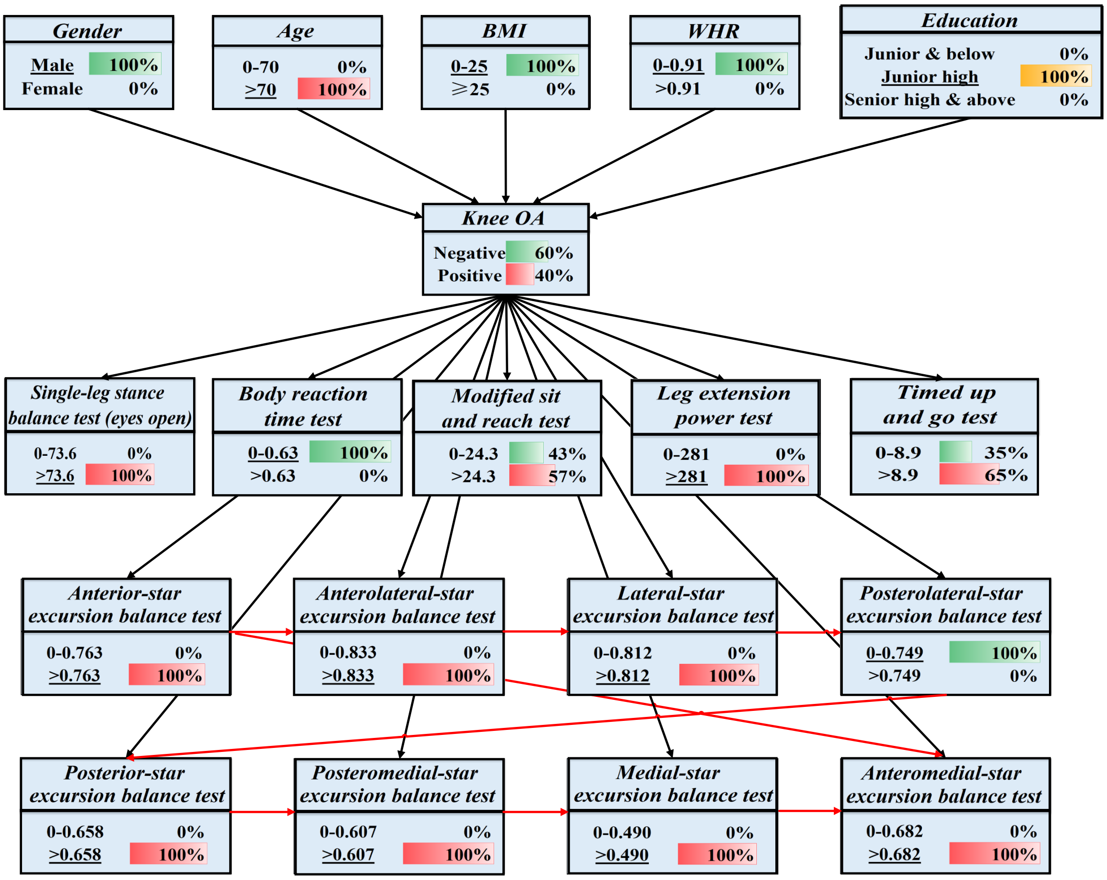

Figure A2. An example of the diagnostic procedure of SHBN model with missing values.
